# Protective effects of Theracurmin treatment during experimental infection of the Colombian strain of *Trypanosoma cruzi* at the testicular site

**DOI:** 10.3389/fcimb.2023.1143360

**Published:** 2023-03-24

**Authors:** Breno Luiz Pimenta, Tatiana Prata Menezes, Vitória Louise, Fernanda Carolina Ribeiro Dias, Bianca Alves Almeida Machado, Lais Ribeiro, Kelerson Mauro de Castro Pinto, Andre Talvani

**Affiliations:** ^1^ Laboratory of Immunobiology of Inflammation, Department of Biological Sciences, Federal University of Ouro Preto, Ouro Preto, Minas Gerais, Brazil; ^2^ Infectious Diseases and Tropical Medicine Post-Graduate Program, School of Medicine, Federal University of Minas Gerais, Belo Horizonte, Minas Gerais, Brazil; ^3^ Health and Nutrition Post-Graduate Program, School of Nutrition, Federal University of Ouro Preto, Ouro Preto, Minas Gerais, Brazil; ^4^ Department of Veterinary, Federal Rural University of Pernambuco, Recife, Pernambuco, Brazil; ^5^ School of Medicine, Federal University of Ouro Preto, Ouro Preto, Minas Gerais, Brazil; ^6^ Laboratory of Physiology of Exercise, School of Physical Education, Federal University of Ouro Preto, Ouro Preto, Minas Gerais, Brazil

**Keywords:** Trypanosoma cruzi, inflammation, testicles, cytokines, theracurmin, Colombian strain

## Abstract

**Introduction:**

Chagas’ disease is a tropical neglected illness caused by Trypanosoma cruzi and remains one of the most significant causes of morbidity and mortality in South and Central Americas. The disease is caused by a moderate to intense and persistent inflammatory response characterized by local upregulated expression and production of inflammatory mediators that favors the activation and recruitment of distinct cells of the immune system into different tissues to eliminate the parasites. Theracurmin is a curcumin’s derived formulation of nanoparticles. Its anti-inflammatory properties make this bioactive compound a mitigating factor in pathological cases after an overwhelming inflammatory response.

**Methods:**

Our research focused on the testicular investigation in 28 mice infected by 103 trypomastigote forms of Colombian strain of T. cruzi and preventively treated with Theracurmin. The mice were treated with 30 mg/Kg of Theracurmin during the period of 30 days. At the 30th day post infection animals were euthanized, and its testicles were collected to morphological and immunological assays.

**Results:**

The animals infected and treated with Theracurmin presented a reduction in the testicular levels of IL-15 and IL-6. The volume density (%) of the tunica propria was also higher in all infected animals, but Theracurmin decreased this parameter in the treated animals. In the intertubular area, the percentage of some intertubular components was decreased in the infected animals such as the percentage and volume of Leydig cells, connective tissue, and macrophages.

**Discussion:**

Furthermore, our data pointed to the daily use of Theracurmin in the diet as a protective element of the testicular function.

## 1 Introduction

Chagas disease is caused by the flagellated protozoan *Trypanosoma cruzi* and affects 6 to 7 million people around the world (World Health Organization, 2022), being endemic from the south of Argentina and Chile though the north of Mexico (Pérez-molina and Molina, 2018). This parasite has several routes of infection, and its main routes are oral and vectorial (during the repast of the triatomine insect while feeding), beyond another ways of transmission such as blood transfusion (mainly in non-endemic countries), laboratory accidents, congenital and sexual transmission, which is supported by experimental studies (Pérez-molina and Molina, 2018; Almeida et al., 2019; Zapparoli et al., 2022).

In the rodents, *T. cruzi* has a different tissue tropism after its infection depending on factors such as the load and genetic background of the parasites and the genetic/immune response related to the mammalian host (Andrade et al., 2010; Medina et al., 2018). The *T. cruzi* tissue tropism is described as heart, skeletal muscle, and tissues from the gastric system (Esper et al., 2015; Weaver et al., 2019; Hossain et al., 2020). However, this parasite infects a diverse range of tissues, including those from reproductive tract (Almeida et al., 2019). However, in animals, *T. cruzi* was found in the testicles and its associated components, such as vas deferens, epididymis, seminal vesicle, prostate, and penis in the acute phase of the experimental infection (Lenzi et al., 1998) and, sexual transmission was described in mice by Martin et al. (2015). These studies opened new questions concerning the capacity of *T. cruzi* to surpass the male reproductive epithelial tissue and induce infection in the female organism. In the reproductive tissue, *T. cruzi* infects cells and cause an inflammation responsible for damages on the reproductive condition and/or the ease of dissemination of the protozoan among infected individuals.

The infection’s treatment using nitrocompounds such as benznidazole is more effective during the acute than in chronic phase and, this effect is also dependent on the genetic background of the protozoan and of the host, which defines its virulence, infectivity and pathogenesis (Cunha-Neto and Chevillard, 2014; Zingales and Bartholomeu, 2022). Since most of the cases are diagnosticated during the chronic phase and, at this time, the trypanocidal effectiveness of benznidazole is partial without reduction of cardiac clinical disturbances (Morillo et al., 2015), studies with new potential drugs or compounds are desirable to the development of a preventive and/or curative treatment. Potential natural/chemical compounds have been investigated to control parasite and the immune response in different infected tissues (De Paula Costa et al., 2016; Menezes et al., 2020).

Curcumin is a polyphenol compound of saffron and it has antioxidant, anti-inflammatory, antiangiogenic, anticarcinogenic and apoptosis regulative properties (Arbiser et al., 1998; Aggarwal et al., 2006; Kuttan et al., 2007; Chiu et al., 2009; Kang and Chen, 2009; Nagajyothi et al., 2013). Curcumin regulates different biological and molecular routes, modulating the synthesis of cytokines, chemokines, enzymes, genes, and transcription factors in distinct inflammatory conditions (Gupta et al., 2012; Gupta et al., 2013; Nakagawa et al., 2020). The use of curcumin as a therapeutic compound in the experimental infection of *T. cruzi* in murine model demonstrated to be effective inhibiting the cellular invasion regulating the LDL receptors in those cells and decreasing the parasitic load in the heart and liver (Yuan et al., 2008; Kang and Chen, 2009; Nagajyothi et al., 2013). Whereas curcumin has a low bioavailability, a new formulation was developed (Theracurmin) whose absorption was 27 higher than curcumin in humans (Nakagawa et al., 2020).

The present study aims to investigate the parasitological and inflammatory patterns in the testicular site of mice infected by the Colombian strain of the *T. cruzi* under preventively treatment with the new formulation of the Theracurmin.

## 2 Materials and methods

### 2.1 Ethical approval

All the methodologies performed in this study were in accordance with the standards of the National Council for Control of Animal Experimentation (CONCEA) and previously approved by the Animal Research Ethics Committee (CEUA) of the Federal University of Ouro Preto (UFOP), Ouro Preto, Minas Gerais, Brazil, under the protocol number 4487110520 (ID 000412).

### 2.2 *Trypanosoma cruzi* infection

For these experiments, we used the Colombian strains of *T. cruzi*, classified as *T. cruzi* I (Zingales, 2009). These strains were maintained by successive passages in Swiss mice at the Center of Animal Science, UFOP.

### 2.3 Animals, *Trypanosoma cruzi* infection and study design

Swiss male mice aged 7-9 weeks weighing approximately 20-40 g were used in this study. Animals (n = 28) were grouped as (i) uninfected (n=7), (ii) uninfected + Theracurmin (n=7), (iii) *T. cruzi* (n=7), and (iv) *T. cruzi* + Theracurmin (n=7). Animals were infected by an intraperitoneal injection of the Colombian strain of the parasite (1000 trypomastigotes/animal). Blood parasites were daily evaluated in infected mice according to Brener’s method (Brener, 1962). On day 30 of infection, the animals were euthanized, their testicles were removed and weighed. The left testes were collected for the immune assay and the right were process to histopathology analysis. The mice were housed and maintained at the Center of Animal Science at UFOP at climatized room with controlled luminosity conditions and temperature (22 ± 2°C).

### 2.4 Theracurmin treatment

One day before the infection, animals were submitted with a daily therapy, *via* gavage, with 30 mg/kg (Sasaki et al., 2011) of Theracurmin (CurcuminRich*^®^*, Natural Factors, Canada) during 30 days. Each capsule of the product had 30 mg of Theracurmin*^®^* (Theravalues, Tokyo, Japan). The individual dose preparation consisted in Theracurmin’s dilution in distilled water with 0.5% of carboxymethyl cellulose. Each animal received 300 μL of this mix and the non-treated groups received the solution vehicle at the same amount.

### 2.5 Testicle processing and histological analysis

Testicular samples of all animals were fixed in tamponed formalin for 24 h, dehydrated in ethanol, embedded in glycol methacrylate resin, and cut into 3 μm thick sections using glass knives (Leica Biosystems, Wetzlar, Germany). Histological slides collected in a semi-series were obtained using one out of every 40 sections to avoid evaluating the same histological area. The sections were stained with toluidine blue.

Digital images obtain with the photomicroscope of light field (Leica DM5000B, Germany) equipped with a digital camera (Leica MC170HD, Germany) were used for morphometric analysis. All images were analyzed using the Image J^®^ software (National Institute of Health, USA).

### 2.6 Parameters of the tubules seminiferous and of the intertubular area

The volumetric proportions (%) of the tubular and intertubular area were estimated with the count of 266 dots in 10 aleatory fields, totaling 2660 dots for each animal in histological images captured with the 10x objective (Dias et al., 2019). The volume (mL) of each testicular component were estimated considering the percentage obtained multiplied by the testicular parenchyma volume. The volume of each component was estimated from the knowledge of its percentage within the testis and the knowledge of the testicular parenchyma volume. Since the mammalian testis density is around 1 (Tae et al., 2005), its weight was considered as the same as the volume. The volumetric proportions (%) of the intertubular components were estimated with the count of 1000 dots projected in captures images of the intertubular area with the 40x objective in different histological slides of each animal (Mouro et al., 2018). The quantified elements were connective tissue, macrophages, blood vessels, lymphatic space and Leydig cells (nucleus and cytoplasm). To calculate the relation between nucleus and cytoplasm of the Leydig cells, the percentage occupied by nucleus was divided by the percentage occupied by cytoplasm (Mouro et al., 2018). The volume (mL) of each intertubular component by the testis was calculated using the following formula: proportion of the element on the testis/(100 × parenchymal mass of one testis) (Russell et al., 1993).

### 2.7 Leydig cell parameters

The diameters of 30 Leydig cells nuclei were measured in each animal, choosing those with circular outline, perinuclear chromatin, and evident nucleoli. To calculate nuclear volume (NV), cytoplasmic volume (CV) and each Leydig cell volume (LCV, the following formulas were used: NV = 4/3 πR3 (R = nuclear radius); CV = % of cytoplasm x NV/% of nucleus; LCV = NV + CV (Russell et al., 1993).

The calculation of the volume that Leydig cells occupy in the testis was performed from the proportion of Leydig cells in the testicular parenchyma x parenchyma weight of one testicle/100. The volume that the Leydig cells occupy per gram of testis was obtained using the gross weight of the testicles. The total number of Leydig cells in the testes was calculated from the volume that the Leydig cells occupy in the testes (μm^3^)/volume of one Leydig cell (μm^3^). To calculate the total number of Leydig cells per gram of testis the following formula was used: volume that the Leydig cell occupies per gram of testis (μm^3^)/volume of one Leydig cell (μm^3^). The Leydigosomatic Index (LSI) was calculated by the formula: LSI = total volume of Leydig cell in the testicular parenchyma/BW x 100 (BW = body weight).

### 2.8 Immunoassay

Levels of CCL2, CXCL10, IL-6 and IL-15 were detected in the supernatant of the homogenized testicular tissues. For sample preparation, 30 mg of testicular tissues were macerated in 300 mL of phosphate buffered saline (PBS) and, after centrifugation at 13000 rpm, for 10 min at 4^°^C, the supernatant was collected (Souza et al., 2021). Inflammatory mediators were measured by enzyme-linked immunosorbent assay (ELISA) using a specific kit (Peprotech, NJ, USA) and were performed according to the manufacturer’s information. The absorbance values were measured using the Biotek *ELx808* (California, USA) ELISA reader at 450 nm.

### 2.9 Statistical analysis

Data are expressed as mean ± standard error of means. Multiple groups were compared using one-way analysis of variance (ANOVA) followed by the Tukey-Kramer post-test. Non paired t test was used to evaluate two independent samples for parametric data. All analyses were performed using the Prism 8 software (GraphPad Software). Groups were considered statistically different at p < 0.05.

## 3 Results

**Figure 1:**
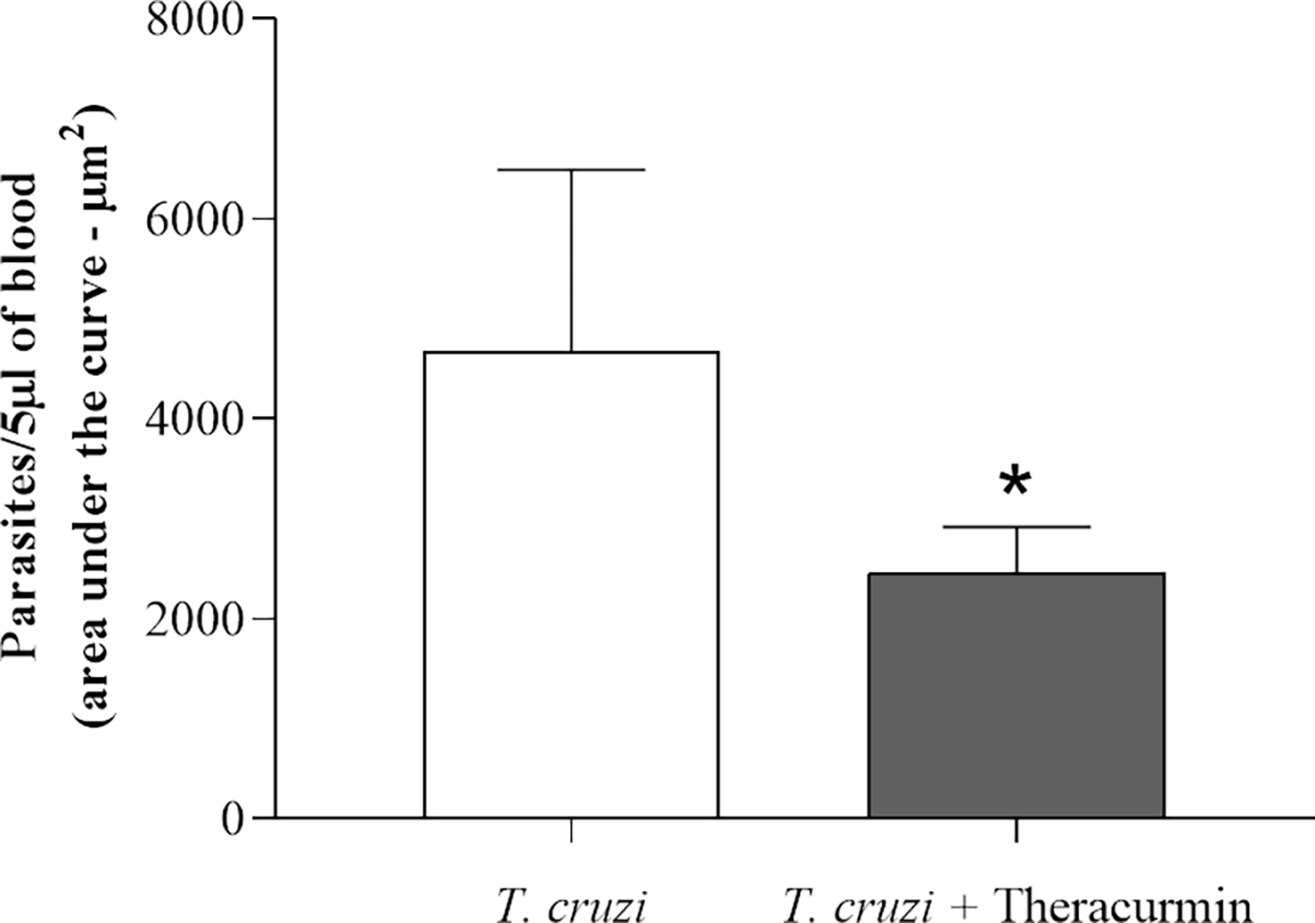
Area under the curve of the parasitemia of the T. cruzi infected group (n=7) and the T. cruzi + Theracurmin group (n=7). Data are expressed as mean ± SE. * shows significant difference (p = 0,0086).

Animals infected with *T. cruzi* and preventively treated with Theracurmin showed a significant reduction in the parasitic load of the circulating blood at the final of the treatment. **Figure 1** represents the area under the curve indicating a significative difference between the circulating *T. cruzi* in both infected groups.

Biometric data obtained from uninfected and *T. cruzi*-infected animals under treatment with Theracurmin is shown in **Table 1**. There was no difference at the body weight of the mice, such as at the albuginea’s weight. In contrast, the testicular weight and gonadosomatic index increased in uninfected animals under Theracurmin treatment when compared to those infected animals that received Theracurmin.

**Table 1:** Biometric and testicular parameters of mice infected with *Trypanosoma cruzi* and treated with Theracurmin.

Parameters	Uninfected	Uninfected + Theracurmin	*T. cruzi*	*T. cruzi +* Theracurmin
BW (g)	42,64±0,39	44,27±2,27	46,19±1,94	45,79±3,38
TW (g)	0,46±0,02**^ab^**	0,55±0,04**^b^**	0,53±0,03**^ab^**	0,42±0,05**^a^**
AW (g)	0,022±0,01	0,025±0,01	0,023±0,01	0,019±0,01
PW (g)	0,43±0,02	0,52±0,04	0,50±0,03	0,40±0,05
GSI (%)	1,07±0,06**^ab^**	1,23±0,06**^b^**	1,14±0,05**^ab^**	0,91±0,10**^a^**
PSI (%)	1,02±0,06**^ab^**	1,18±0,06**^b^**	1,09±0,0**^ab^**	0,87±0,10**^a^**

BW, Body weight; TW, Testicular weight; AW, Albuginea’s weight; PW, Parenchyma’s weight; GSI, Gonadosomatic index; PSI, Parenchyosomatic index. Data expressed as mean ± standard error (SE). Different letters between groups shows significant differences (p≤0,05).

**Figure 2:**
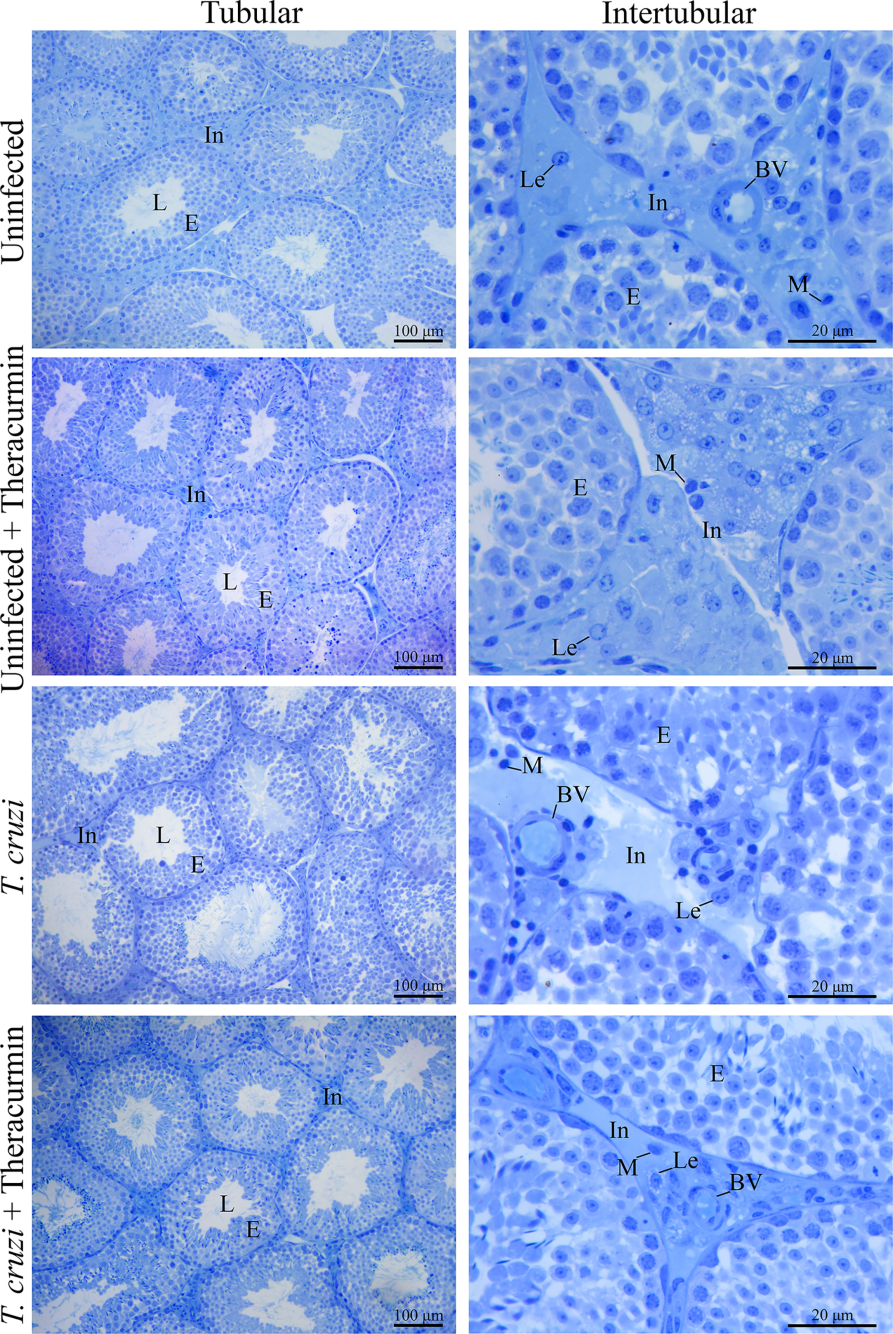
Histological images of testicles of Swiss mice infected with *T. cruzi* and treated with Theracurmin. Animals (n = 28) were grouped as (i) uninfected (n=7), (ii) uninfected + Theracurmin (n=7), (iii) *T. cruzi* (n=7), and (iv) *T. cruzi* + Theracurmin (n=7). At the second day after the beginning of the treatment, mice were intraperitoneally infected with 1000 trypomastigote forms of Colombian strain of *T. cruzi*. After 30 dpi testicles were fixed, embedded in glycol methacrylate resin, and cut into 3 mm thick sections. The sections were stained with toluidine blue. The volumetric proportions of the tubular and intertubular area were estimated with the count of 266 dots in 10 aleatory fields and, the volumetric proportions of the intertubular components were estimated with the count of 1000 dots projected in captured images of the intertubular area in different histological slides of each animal. In, Intertubular area; L, Lumen; E, Epithelium; Lc, Leydig cell; BV, Blood vessel; M, Macrophage.

In the histological analysis, there was not observed the presence of the amastigote nests in testicles, in the tubular, neither in the intertubular areas (**Figure 2**). In the morphometric analysis, there was an increase in the percentage of the tubules seminiferous and of the tunica propria in *T. cruzi*-infected animals when compared to those without infection, independent of the treatment with Theracurmin. However, in the infected groups under Theracurmin treatment, we observed a decrease in the percentage of the tunica propria when compared to the animals without the treatment. In addition, the tubulosomatic index raised in the uninfected animals under treatment and the Theracurmin was able to decrease the epitheliumsomatic index in the animals infected by *T. cruzi* (**Table 2**).

**Table 2:** Parameters of the tubules seminiferous of Swiss mice infected with T. cruzi and treated with Theracurmin.

	Uninfected	Uninfected + Theracurmin	*T. cruzi*	*T. cruzi +* Theracurmin
TS (%)	87,17±0,85**^a^**	89,40±0,85**^ab^**	93,29±0,36**^b^**	90,43±1,66**^b^**
EP (%)	78,01±1,16	79,04±1,35	80,73±1,22	76,49±1,97
TP (%)	1,50±0,08**^a^**	1,88±0,11**^a^**	3,04±0,07**^b^**	2,45±0,23**^c^**
L (%)	7,66±0,67	8,47±0,77	9,52±1,35	11,48±1,74
TSI (%)	0,89±0,05**^ab^**	1,05±0,06**^c^**	1,01±0,04**^ac^**	0,79±0,10**^b^**
ESI (%)	0,80±0,05**^ab^**	0,93±0,06**^a^**	0,88±0,04**^a^**	0,67±0,09**^b^**

TS, Tubules seminiferous; EP, Epithelium; TP, Tunica propria; L, Lumen; TSI, Tubulosomatic index; ESI, Epitheliumsomatic index. Data expressed as mean ± SE. Different letters between groups shows significant differences (p≤0,05).

The stereological analysis of the tubules seminiferous demonstrated an increase at the luminal diameter and luminal area in the animals infected by *T. cruzi*. Therefore, the tubules/epithelium ratio was higher in infected mice (**Table 3**).

**Table 3:** Stereological analysis of the tubules seminiferous of Swiss mice infected with T. cruzi and treated with Theracurmin.

	Uninfected	Uninfected + Theracurmin	*T. cruzi*	*T. cruzi +* Theracurmin
EH (μm)	96,86±5,59	93,06±3,42	89,45±4,29	83,65±8,30
TD (μm)	265,98±8,28	274,47±12,69	278,19±12,18	253,18±17,67
LD (μm)	72,25±5,04**^a^**	88,35±6,56**^ab^**	99,30±8,20**^b^**	85,88±5,45**^ab^**
TLT (m)	6,91±0,61	8,04±0,81	7,83±0,66	7,04±0,29
TLT/g	15,11±0,99	14,81±1,38	14,98±1,15	18,31±2,78
Tubular area	55765,93±3490,96	59676,77±5629,25	61248,95±5504,20	51367,71±6674,58
Luminal area	4183,18±550,10**^a^**	6272,12±945,39**^ab^**	7965,69±1206,71**^b^**	5889,90±774,06**^ab^**
Epithelial area	51582,76±3707,49	53404,65±4740,44	53283,26±4746,24	45477,81±6411,40
TER	1,08±0,01**^a^**	1,12±0,01**^ab^**	1,15±0,02**^b^**	1,14±0,03**^ab^**

EH, Epithelium height; TD, Tubular diameter; LD, Luminal diameter; TLT, Total length of the tubules seminiferous; TLT/g, Total length of the tubules seminiferous per grama of testicle; TER, Tubules/epithelium ratio. Data expressed as mean ± SE. Different letters between groups shows significant differences (p≤0,05).

At the intertubular area, its percentual reduced significantly in the presence of *T. cruzi* and *T. cruzi* + Theracurmin when compared to the non-infected animals, while the Leydig nucleus percentage reduced only at the infected animals in comparison those uninfected. The Leydig cytoplasm and de Leydig cell percentages were decreased in the presence of *T. cruzi*, while the percentage of connective tissue was decreased in the presence of *T. cruzi* plus treatment with Theracurmin. The macrophages percentage were also smaller in the presence of *T. cruzi* when compared to the uninfected and untreated group (**Table 4**).

**Table 4:** Percentage and volume of the intertubular components in mice infected with T. cruzi and treated with Theracurmin.

	Uninfected	Uninfected + Theracurmin	*T. cruzi*	*T. cruzi +* Theracurmin
IT (%)	12,83±0,85**^a^**	10,60±0,85**^ab^**	6,71±0,36**^b^**	9,57±1,66**^b^**
BV (%)	0,88±0,24	0,59±0,10	0,50±0,18	0,52±0,15
LS (%)	0,78±0,19	1,29±0,43	1,43±0,32	1,93±0,41
LN (%)	1,36±0,13**^a^**	0,94±0,09**^ab^**	0,66±0,06**^b^**	1,08±0,19**^ab^**
Lcit (%)	8,23±1,30**^a^**	7,31±0,85**^a^**	3,89±0,37**^b^**	5,53±0,94**^ab^**
LC (%)	9,58±1,41**^a^**	8,26±0,91**^a^**	4,54±0,41**^b^**	6,61±1,12**^ab^**
CT (%)	1,29±0,48**^a^**	0,29±0,08**^b^**	0,12±0,02**^b^**	0,28±0,09**^b^**
MAC (%)	0,31±0,09**^a^**	0,18±0,05**^ab^**	0,12±0,01**^b^**	0,24±0,04**^ab^**
IT (mL)	0,0558±0,01**^ac^**	0,0543±0,01**^c^**	0,0336±0,01**^b^**	0,0359±0,01**^ab^**
BV (mL)	0,0039±0,0012	0,0030±0,0004	0,0026±0,0010	0,0019±0,0005
LS (mL)	0,0035±0,0009	0,0071±0,0026	0,0073±0,0018	0,0072±0,0014
LN (mL)	0,0058±0,0,0005**^a^**	0,0048±0,0004**^ab^**	0,0033±0,0004**^b^**	0,0040±0,0006**^b^**
Lcit (mL)	0,0354±0,0062**^a^**	0,0370±0,0024**^a^**	0,0192±0,0014**^b^**	0,0209±0,0030**^b^**
LC (mL)	0,0412±0,0066	0,0418±0,0025	0,0225±0,0017	0,0249±0,0035
CT (mL)	0,0058±0,0024**^a^**	0,0014±0,0003**^b^**	0,0006±0,0001**^b^**	0,0010±0,0002**^b^**
MAC (mL)	0,0014±0,0005	0,0009±0,0003	0,0006±0,0001	0,0009±0,0001

IT, Intertubule; BV, Blood vessel; LS, Lymphatic space; LN, Leydig nucleus; Lcit, Leydig cytoplasm; LC, Leydig cell; CT, connective tissue; MAC, Macrophage. Data expressed as mean ± SE. Different letters between groups shows significant differences (p≤0,05).

The intertubular volume (mL) reduced in those animals infected by *T. cruzi*. These animals also had a smaller volume of Leydig nucleus and cytoplasm when compared to the non-infected animals. According to its percentage, the connective tissue volume was decreased in the presence of the parasite plus Theracurmin treatment (**Table 4**).

According to the morphometric and stereological parameters of the Leydig cells, its cytoplasmatic and cellular volumes were reduced in the presence of *T. cruzi* + Theracurmin when compared to the uninfected animals + Theracurmin. Furthermore, the Leydig cellular volume per testicle was reduced in the animals infected by the protozoan, while the Leydig cellular volume per gram of testicle were smaller only at the *T. cruzi* group, when compared to the non-infected animals. The number of Leydig cells per testicle also reduced in the infected animals when compared to the uninfected one. Even though the number of Leydig cells per testicle did not show difference between the infected groups, the Theracurmin treatment inhibit the decrease of the number of Leydig cells per gram of testicle. The leydigosomatic index were also reduced in the infected animals, when compared to those uninfected (**Table 5**).

**Table 5:** Morphometric and stereological parameters of Leydig cells in mice infected with T. cruzi and treated with Theracurmin.

	Uninfected	Uninfected + Theracurmin	*T. cruzi*	*T. cruzi +* Theracurmin
LeydigD (μm)	7,16±0,21	7,07±0,16	6,92±0,19	6,70±0,15
Vol. LN (μm³)	194,35±16,92	186,39±12,89	175,30±15,20	158,21±10,81
Vol. Lcit (μm³)	1179,97±213,51**^ab^**	1473,83±200,47**^b^**	1035,37±83,88**^ab^**	822,62±77,49**^a^**
Vol. LC(μm³)	1374,32±227,61**^ab^**	1660,22±209,05**^b^**	1210,67±92,21**^ab^**	980,83±85,95**^a^**
Vol. LC/t (mL)	0,041±0,01**^a^**	0,042±0,00**^a^**	0,022±0,00**^b^**	0,025±0,00**^b^**
Vol. LC/t (μm³)	4,1x10^10^±6,6x10^9^ **^a^**	4,2x10^10^±2,5x10^9^ **^a^**	2,2x10^10^±1,7x10^9^ **^b^**	2,5x10^10^±3,5x10^9^ **^b^**
Vol. LC/gt (mL)	0,091±0,01**^a^**	0,078±0,01**^a^**	0,043±0,00**^b^**	0,063±0,01**^ab^**
Vol. LC/gt (μm³)	9,1x10^10^±1,3x10^10^ **^a^**	7,8x10^10^±8,0x10^9^ **^a^**	4,3x10^10^±3,7x10^9^ **^b^**	6,3x10^10^±1,1x10^10^ **^ab^**
LCn/t	3,0x10^7^±2,6x10^6^ **^a^**	2,7x10^7^±3,3x10^6^ **^ab^**	1,9x10^7^±1,5x10^6^ **^b^**	2,5x10^7^±2,9x10^6^ **^ab^**
LCn/gt	6,7x10^7^±5,6x10^6^ **^a^**	4,9x10^7^±6,0x10^6^ **^ab^**	3,6x10^7^±3,4x10^6^ **^b^**	6,4x10^7^±8,2x10^6^ **^a^**
LSI (%)	0,097±0,02**^a^**	0,095±0,01**^a^**	0,049±0,01**^b^**	0,054±0,01**^b^**

LeydigD, Leydig diameter; LN, Leydig nucleus; Lcit, Leydig cytoplasm; LC, Leydig cell; LCn, Leydig cell number; t, testicle; g, gram; LSI, Leydigosomatic index. Data expressed as mean ± SE. Different letters between groups shows significant differences (p≤0,05).

**Figure 3:**
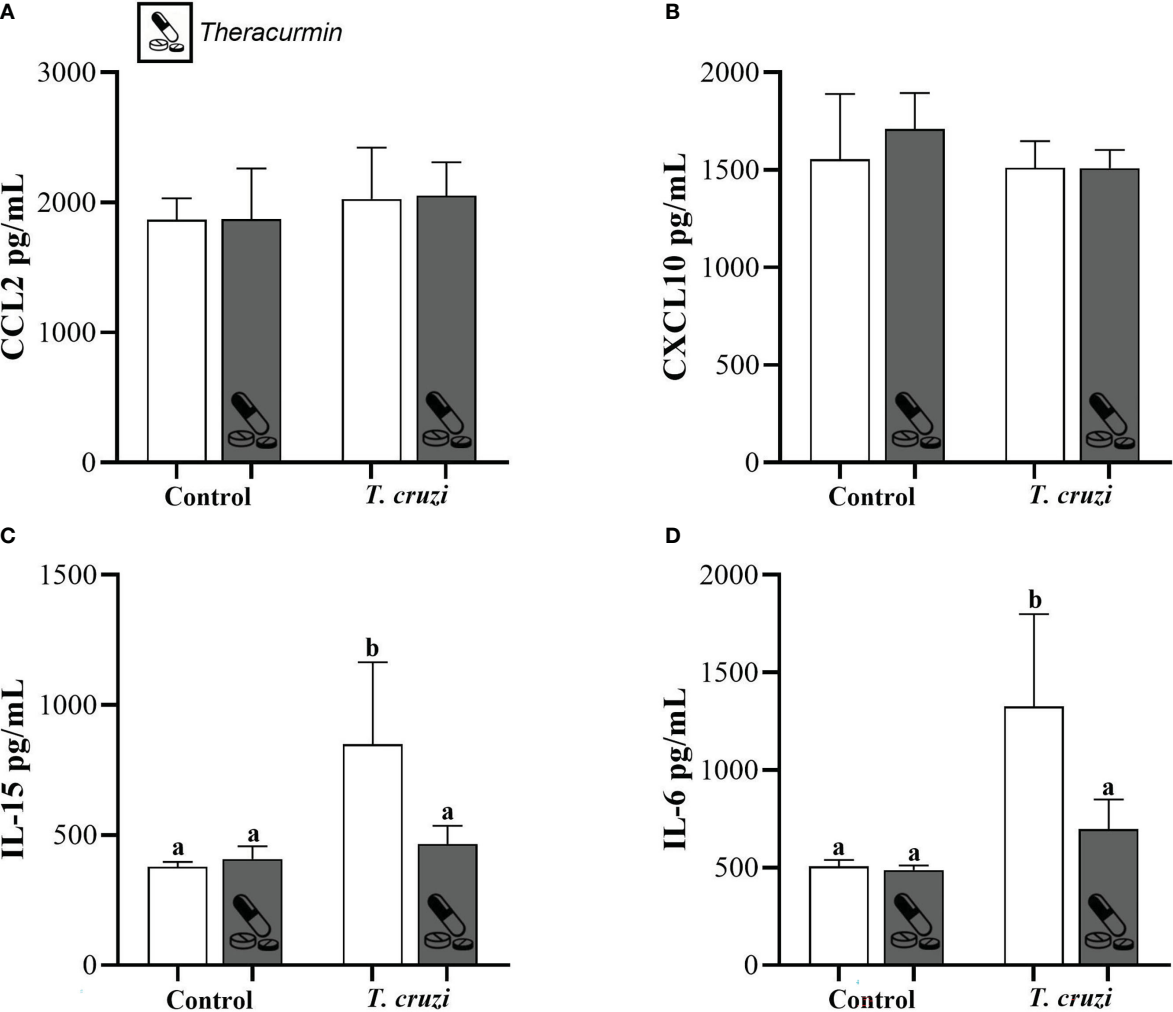
Production of CCL2 **(A)**, CXCL10 **(B)**, IL-15 **(C)**, and IL-6 **(D)** at testicles of Swiss mice infected with T. cruzi and treated with Theracurmin. Animals (n = 28) were grouped as (i) uninfected (n=7), (ii) uninfected + Theracurmin (n=7), (iii) *T. cruzi* (n=7), and (iv) *T. cruzi* + Theracurmin (n=7). At the second day after the beginning of the treatment, mice were intraperitoneally infected with 1000 trypomastigote forms of Colombian strain of *T. cruzi*. After 30 dpi the concentration of these cytokines was evaluated by the enzymatic immunoassay method at the testicular macerated. Data expressed as mean ± SE. Different letters between groups shows significant differences (p ≤ 0,05).

Finally, the concentration of CCL2, CXCL10 did not suffer any alterations in the animals infected with *T. cruzi* and/or treated with Theracurmin with in the testicular site (**Figures 3A, B**). Despite of that, the concentration of IL-15 and IL-6 shows that the *T. cruzi* have increased the production of those inflammatory markers, but the animals treated with Theracurmin were capable of reduce these cytokine levels in testicular area (**Figures 3C, D**).

## 4 Discussion

This present study aimed to verify if the preventive treatment with Theracurmin (highly bioavailable curcumin) was able to regulate the morphological and/or inflammatory parameters caused by the infection of *T. cruzi* in experimental model. Previously, curcumin was able to reduce the parasitemia in *T. cruzi* infected animals (Nagajyothi et al., 2013) which is explained by the inhibitory effect of curcumin at the transcription of low-density lipoprotein receptor (LDLr) (Yuan et al., 2008; Kang and Chen, 2009; Nagajyothi et al., 2013) as it is known that the *T. cruzi* utilizes these LDLr during its cellular invasion process (Nagajyothi et al., 2011).

The Colombian strain of *T. cruzi* has a positive tropism for muscular tissues, infecting myoid cells frequently (Melo and Brener, 1978; Camandaroba et al., 2001; Andrade et al, 2010). Research conducted by Carvalho et al. (2009) showed that the protozoan can infect the tunica propria cells of the tubule seminiferous and may have that preference because of contractible proteins responsible for the movement of non-motile sperm cells throughout the tubules (Lenzi et al., 1998). At this present study, we found a higher volumetric percentage and volume of the tunica propria cells in the presence of the *T. cruzi*, even though we did not find amastigotes of the parasite in the testicular site. Theracurmin may have a protective role in muscular-like cells and tissue (Hernández et al., 2021), resulting in a reduced cellular hyperplasia at the testicular tunica propria in treated mices. The infection by *T. cruzi* also induced a significant raise in the luminal diameter and area of the tubules seminiferous resulting in a higher tubule-epithelial ratio. The tubules seminiferous are composed of three main components: tunica propria, epithelium and lumen, this last structure is where the spermatozoa are released after spermatogenesis (Ross, 1974). This difference at the lumen may be the result of a compensatory effect due to the inflammatory stress caused by *T. cruzi* infection.

At this present study we found significant decrease in components of the intertubular area, such as the percentage of resident macrophages and the percentage and volume of the connective tissue during *T. cruzi* infection, corroborating the data in Mendis-Handagama et al. (1990), suggesting an alteration of the intertubular site caused by the inflammation at testicular site. The treatment with Theracurmin also led to a decrease in the percentage and volume of connective tissue probably because of the inhibition generated by the treatment in the genic expression of growth factors at the connective tissue causing a reduction at the extracellular matrix including type I collagen and fibronectin (Xu et al., 2003; Chen and Zheng, 2008).The Leydig cells and resident macrophages of the intertubular area of the testicles has an intimate relation as these macrophages are responsible for the release of biomarkers that affect directly at the diametric growth and development of the testosterone producers’ cells (Hales, 2007). In this context, the reduced presence of resident macrophages, as observed in the present study, may result in a subdevelopment of the Leydig cells. The *T. cruzi* is a crucial element to potentialize the local inflammatory response with its immunogenic molecules and, even in environment without inflammation, macrophages release important factors that contribute to the development of Leydig cells (Hales et al., 1999; Hales, 2002). The decreased in Leydig cell population may be due to an intense inflammation generated by the *T. cruzi*, reducing progressively the genic expression and function of these cells, as showed by Aldahhan et al. (2021) in rats with cryptorchids.

The infection caused by the protozoan *T. cruzi* is well known to cause an intense inflammatory response in a diverse range of tissues in the mammal host body with the release of inflammatory markers, such as cytokines and chemokines (Talvani and Teixeira, 2011). The curcumin has an anti-inflammatory and protective effects in tissues such as the heart and liver during the stress associated with chronic diseases, preserving their function, and regulating the release of inflammatory markers (Nagajyothi et al., 2013; Hernández et al., 2021). At the macerated of the testicles, Theracurmin treatment was able to reduce the concentration of the proinflammatory cytokines IL-6 and IL-15. Produced by the Sertoli cells, IL-6 has an important role at paracrine/autocrine regulation during spermatogenesis and steroidogenesis and presenting in higher concentrations during exacerbated inflammation (Hedger and Meinhardt, 2003; Rival et al., 2006). IL-15 has also presented higher concentration during microorganisms’ infection and may have an important role in innate and adaptative response at testicular site (Anastasiadou and Michailidis, 2016). Even though the concentrations of CCL2 and CXCL10 are normally higher in heart and blood during *T. cruzi* infection (De Araújo et al., 2020), our data did not show difference at testicular site. This fact reinforce that the inflammatory response conducted by the *T. cruzi* is compartmentalized and, in parts, defined by the genetic aspects of the parasite and its studied host.

In conclusion, our study demonstrated that the Colombian strain of the *T. cruzi* may cause immunological and punctual structural alterations at the testicular site in mice infected, mainly at the tunica propria, muscle layer that recovers the tubules seminiferous, and at the Leydig cells. Moreover, the preventive treatment with Theracurmin presented protective effects, regulating the synthesis of IL-6 and IL-15, but not CCL2 and CXCL10, in the testicular area. The relation between the *T. cruzi* infection and the testosterone production at the Leydig cells still needs to be researched for a bigger understanding of the damage caused by the protozoan at the testicular site and the relation with the spermatogenesis.

## Data availability statement

The raw data supporting the conclusions of this article will be made available by the authors, without undue reservation.

## Ethics statement

It was approved by the Animal Research Ethics Committee (CEUA) of the Federal University of Ouro Preto (UFOP), Ouro Preto, Minas Gerais, Brazil, under the protocol number 4487110520.

## Author contributions

The authors’ contributions were as follows: BP, TM, AT: conception, design, writing and final content; VS, FD, BM, LR and BP: perform the experiments; KP, TM, BP and AT: data analysis; AT: funding acquisition. All authors contributed to the article and approved the submitted version.
